# Recursive Random Lasso (*RRLasso*) for Identifying Anti-Cancer Drug Targets

**DOI:** 10.1371/journal.pone.0141869

**Published:** 2015-11-06

**Authors:** Heewon Park, Seiya Imoto, Satoru Miyano

**Affiliations:** Human Genome Center, Institute of Medical Science, University of Tokyo, Tokyo, Japan; University of Miami, UNITED STATES

## Abstract

Uncovering driver genes is crucial for understanding heterogeneity in cancer. *L*_1_-type regularization approaches have been widely used for uncovering cancer driver genes based on genome-scale data. Although the existing methods have been widely applied in the field of bioinformatics, they possess several drawbacks: subset size limitations, erroneous estimation results, multicollinearity, and heavy time consumption. We introduce a novel statistical strategy, called a Recursive Random Lasso (*RRLasso*), for high dimensional genomic data analysis and investigation of driver genes. For time-effective analysis, we consider a recursive bootstrap procedure in line with the random lasso. Furthermore, we introduce a parametric statistical test for driver gene selection based on bootstrap regression modeling results. The proposed *RRLasso* is not only rapid but performs well for high dimensional genomic data analysis. Monte Carlo simulations and analysis of the “Sanger Genomics of Drug Sensitivity in Cancer dataset from the Cancer Genome Project” show that the proposed *RRLasso* is an effective tool for high dimensional genomic data analysis. The proposed methods provide reliable and biologically relevant results for cancer driver gene selection.

## Introduction

Much research is currently underway to understand the complexity of the heterogeneous genetic networks underlying cancer. To identify the heterogeneous genetic networks that underlie cancer, various large scale-omics projects (e.g., The Cancer Genome Project, The Cancer Genome Atlas (TCGA), Sanger Genomics of Drug Sensitivity in Cancer dataset from the Cancer Genome Project, and others) have been initiated and have provided large amounts of data, such as genomic and epigenomic data for cancer patients or cell lines. A crucial issue in cancer research is to identify cancer driver genes based on various genomic data analysis (e.g., expression levels, copy number variations, methylation, and others), since efficient identification of cancer drug targets facilitates development of successful anti-cancer therapies. Although various *L*_1_-type regularization approaches, e.g., lasso [1] and elastic net [2], have been widely used to identify cancer driver genes, they possess several drawbacks as tools for driver gene identification [3]. The lasso and adaptive lasso [4] suffer from the limitation of subset size (i.e., these methods select features at most sample size, *n*). The elastic net, which has been widely used in bioinformatics research, may provide erroneous estimation results for coefficients of highly correlated variables with different magnitudes, especially those that differ in sign, because of its “grouping effect”. However, coefficients of highly correlated variables with different magnitudes are frequently observed in bioinformatics research, since genes in common biological pathways are usually correlated, and their regression coefficients can have different magnitudes or different signs. Furthermore, adaptive *L*_1_-type regularization methods suffer from multicollinearity, since their adaptive data driven weights are based on Ordinary Least squares (OLS) estimators.

To resolve these issues, Wang et al. [3] proposed a random lasso based on bootstrap regression modeling with random forest method. Although the random lasso overcomes the drawbacks of existing *L*_1_-type regularization approaches by using a random forest strategy, the method is computationally intensive because it employs two step bootstrap procedures. Furthermore, Wang et al. [3] performed final feature selection based on an arbitrarily decided threshold, even though the variable selection results heavily depend on the threshold.

We propose a novel statistical strategy to identify driver genes of anti-cancer drug sensitivity in line with the random lasso. We introduce recursive bootstrap approaches to simultaneously measure the significance of each gene and perform driver gene selection. We also propose a novel threshold based on a parametric statistical test to effectively identify driver genes based on bootstrap regression modeling. By using a recursive bootstrap procedure, we perform time-efficient bootstrap regression modeling for high dimensional genomic data analysis without loss of modeling accuracy. Furthermore, the proposed feature selection method using parametric statistical test can be a useful tool for variable selection based on the bootstrap regression modeling.

Using Monte Carlo simulations of various scenarios, we demonstrate the effectiveness of the proposed recursive random lasso and elastic net with a parametric statistical test for high dimensional regression modeling. We also apply the proposed statistical strategy to the publicly available “Sanger Genomics of Drug Sensitivity in Cancer dataset from the Cancer Genome Project” (http://www.cancerrxgene.org/), and identify potential driver genes of anti-cancer drug sensitivity. Numerical analyses show that the proposed recursive random lasso and elastic net are time-efficient procedures, and outperform high dimensional genomic data analysis (i.e., from a view point of feature selection and predictive accuracy).

In Section 2, we introduce the existing *L*_1_-type regularization approaches, and point out their drawbacks. We then introduce the random lasso, and propose the recursive random lasso and elastic net procedures. In Section 3, we describe the Monte Carlo simulations and driver gene selection using the Sanger Genomics of Drug Sensitivity in Cancer dataset to examine the effectiveness of the proposed statistical strategies. We state our conclusions in Section 4.

## Materials and Methods

Suppose we have *n* independent observations {(*y*_*i*_, ***x***_*i*_);*i* = 1, …, *n*}, where *y*_*i*_ are random response variables and **x**_*i*_ are *p*-dimensional vectors of the predictor variables. Consider the linear regression model,

$$\begin{array}{c} y_{i}=x_{i}^{T}\beta +\varepsilon_{i},\;i=1,...,n, \end{array}$$ (1)

where ***β*** is an unknown *p*-dimensional vector of regression coefficients and *ε*_*i*_ are the random errors which are assumed to be independently and identically distributed with mean 0 and variance *σ* ^2^. We assume that the *y*_*i*_ are centered and *x*_*ij*_ are standardized by their mean and standard deviation: $\sum_{i}^{n}y_{i}/n=0$, $\sum_{i}^{n}x_{ij}/n=0$ and $\sum_{i}^{n}x_{ij}^{2}/n=1$, thus an intercept term is excluded from the regression model in Eq (1). Many studies are currently underway on regression modeling, especially for high dimensional data analysis (e.g., genomic alterations data analysis).

Tibshirani [1] proposed the lasso, which minimizes the residual sum of squares subject to a constraint $\lambda \sum_{j=1}^{p}\left|\beta_{j}\right|$, and its solution is given by

$$\begin{array}{c} {\hat{\beta }}^{\text{LA}}=\underset{\beta }{\text{arg}\;\text{min}}\{\sum_{i=1}^{n}{\left(y_{i}-x_{i}^{T}\beta \right)}^{2}+\lambda \sum_{j=1}^{p}|\beta_{j}\left|\right\}, \end{array}$$ (2)

where *λ* is a tuning parameter controlling model complexity. By imposing a penalty term, the sum of the absolute values of the regression coefficients, the lasso can simultaneously perform parameter estimation and variable selection.

However, a recent work suggested that the lasso may suffer from the following limitations [2]:

- In the *p* > *n* case, the lasso selects at most *n* variables, because of the convex optimization problem. This implies that the lasso is not suitable for driver gene selection, since genomic alteration data is typically high dimensional data.
- The lasso cannot account for grouping effect of predictor variables, and thus tends to select only one variable from among highly correlated variables, even if all are related to response variable. However, genomic alterations of genes (e.g., expression levels, copy number variations, methylation, etc.) that share a common biological pathway are usually highly correlated, and the genes may be associated with a complex cancer mechanism considered to be the response variable. This also implies that the lasso is not suitable for genomic data analysis.

To overcome these drawbacks, various *L*_1_-type regularization methods have been proposed. The elastic net [2] in particular has drawn considerable attention in the field of bioinformatics:

$$\begin{array}{c} {\hat{\beta }}^{\text{ELA}}=\underset{\beta }{\text{arg}\;\text{min}}\{\sum_{i=1}^{n}{\left(y_{i}-x_{i}^{T}\beta \right)}^{2}+\lambda_{1}\sum_{j=1}^{p}|\beta_{j}\left|+\lambda_{2}\sum_{j=1}^{p}\beta_{j}^{2}\right\}. \end{array}$$ (3)

The penalty term of the elastic net is a convex combination of the ridge [5] and lasso penalties. By imposing an additional *L*_2_-penalty on the lasso, the elastic net performs effectively feature selection in high dimensional data analysis, i.e., there is no limitation on subset size. Furthermore, the elastic net can enjoy the following grouping effect:

$$\begin{array}{c} D_{\lambda_{1},\lambda_{2}}\left(j,k\right)=\frac{1}{{\left|y\right|}_{1}}\left|{\hat{\beta }}_{j}\left(\lambda_{1},\lambda_{2}\right)-{\hat{\beta }}_{k}\left(\lambda_{1},\lambda_{2}\right)\right|\leq {\frac{1}{\lambda }}_{2}\sqrt{2(1-\rho )}, \end{array}$$ (4)

where $\rho =x_{j}^{T}x_{k}$ is sample correlation [2].

Although the elastic net performs well for high dimensional data analysis, Wang et al. [3] demonstrated that the elastic net has the following drawbacks:

- The property of “grouping effect” leads to erroneous estimation results when coefficients of highly correlated variables with different magnitudes, especially those with different signs. However, coefficients of highly correlated variables with different magnitudes are frequently observed in bioinformatics research, since genes in the common biological pathway are usually highly correlated, and their regression coefficients can have different magnitudes or a different sign.

The adaptive *L*_1_-type penalties have also been proposed and are widely used in various fields of research:

- adaptive lasso:
  $$\begin{array}{c} P_{\lambda }^{\text{Ad}.\text{LA}}\left(|\beta |\right)=\lambda \sum_{j=1}^{p}w_{j}\left|\beta_{j}\right|, \end{array}$$ (5)
- adaptive elastic net:
  $$\begin{array}{c} P_{\lambda }^{\text{Ad}.\text{ELA}}(|\beta |)=\lambda \{\left(1-\alpha \right)\sum_{j=1}^{p}w_{j}|\beta_{j}\left|+\alpha \sum_{j=1}^{p}\beta_{j}^{2}\right\}, \end{array}$$ (6)

where $w_{j}=1/{\left|{\hat{\beta }}_{j}^{OLS}\right|}^{\gamma }$ is an adaptive data driven weight for *γ* > 0. By using the weight, we can discriminately impose a penalty on each feature depending on their significance, and thus effectively perform feature selection. Zou and Hastie [4] and Zou and Zhang [2] established the oracle property of the adaptive lasso and the adaptive elastic net, respectively. However, the performance of adaptive regularization methods heavily depends on the OLS estimator, and thus these methods suffer from multicollinearity. Furthermore, the adaptive *L*_1_-type regularization methods suffer from the same drawbacks as the common methods, i.e., when using the adaptive lasso, the number of selected variables cannot exceed *n*, and the adaptive elastic net may also provide erroneous estimation results when coefficients of highly correlated variables with different magnitudes are present.

### Random Lasso

Wang et al. [3] detailed the drawbacks of existing *L*_1_-type approaches, and proposed the random lasso based on a bootstrap strategy that employs the random forest method. In the random lasso procedure, randomly selected *q* variables are considered as candidate variables in regression modeling for each bootstrap sample. Thus, the results do not suffer from the highly correlated variables drawbacks, since each bootstrap sample may include only a subset of the highly correlated variables. Furthermore, the random lasso can overcome the subset size limitation, since variable selection is based on the results of bootstrap regression modeling with randomly selected *q*_1_ or *q*_2_ variables in each bootstrap sample.

Wang et al. [3] proposed the following algorithm based on a two-step bootstrap procedure to implement the random lasso:

**Algorithm 1** *Random lasso*

- Step 1: Generating importance measures of predictor variables.
  ∘ Draw *B* bootstrap samples with size *n* by sampling with replacement from the original dataset.
  ∘ For the $b_{1}^{th}$ bootstrap sample, *b*_1_ ∈ {1, 2, …, *B*}, *q*_1_ candidate variables are randomly selected, and the lasso is applied for regression modeling and we obtain estimators ${\hat{\beta }}_{j}^{\left(b_{1}\right)}$ for *j* = 1, …, *p*.
  ∘ The importance measure of *x*_*j*_ is calculated as $I_{j}=\left|B^{-1}\sum_{b_{1}=1}^{B}{\hat{\beta }}_{j}^{\left(b_{1}\right)}\right|$.
- Step 2: Variable selection
  ∘ Draw *B* bootstrap samples with size *n* by sampling with replacement from the original dataset.
  ∘ For the $b_{2}^{th}$ bootstrap sample, *b*_2_ ∈ {1, 2, …, *B*}, *q*_2_ candidate variables are randomly selected with a selection probability of *x*_*j*_ proportional to *I*_*j*_, and the adaptive lasso is applied for regression modeling, and we obtain the estimator ${\hat{\beta }}_{j}^{\left(b_{2}\right)}$ for *j* = 1, …, *p*.
  ∘ Compute the final estimator, ${\hat{\beta }}_{j}$, as ${\hat{\beta }}_{j}=B^{-1}\sum_{b_{2}=1}^{B}{\hat{\beta }}_{j}^{\left(b_{2}\right)}$ for *j* = 1, …, *p*.

For noise predictor variables, the coefficients in the respective bootstrap samples are estimated to be small or to have different signs, and thus the absolute value of the average coefficients (i.e., *I*_*j*_) will be small or close to zero. On the other hand, the coefficients of crucial predictor variables may be consistently large in different bootstrap samples, and thus a crucial gene has a large value of *I*_*j*_. This implies that the selection probability *I*_*j*_ provides effective feature selection. Wang et al. [3] considered *q*_1_ and *q*_2_ as tuning parameters, and the importance measure *I*_*j*_ can also be used to weight for the adaptive lasso.

Wang et al. [3] noted that the variable selection results of the random lasso are unfair, since some of the final non-zero coefficients may result from a particular bootstrap sample (i.e., the random lasso can yield false positives in variable selection). Thus, a threshold *t*_*n*_ = 1/*n* was added for variable selection, and predictor variables with $|{\hat{\beta }}_{j}|⩽\;t_{n}$ were deleted from the final model.

### Recursive Random Lasso for Effective Feature Selection

The random lasso can overcome the drawbacks of existing *L*_1_-type regularization by using a random forest method with bootstrap regression modeling. Although the random lasso performs well for high dimensional regression modeling with highly correlated predictors, the method also suffers from the following drawbacks:

- The random lasso is computationally intensive, since it is based on two bootstrap procedures with respective B replications. The computational complexity of the random lasso is significantly increased in genomic data analysis, because the dataset is constructed with an extremely large number of predictor variables.
- The threshold is crucial in feature selection, since the feature selection results depend heavily on the threshold. However, Wang et al. [3] arbitrarily set the threshold as 1/*n*, without any statistical background.
- The method has too many tuning parameters, i.e., *λ* in *L*_1_-type penalties, and *q*_1_ and *q*_2_ in the random forest method. The large number of tuning parameters also makes the method time consuming, since the random lasso procedures should be implemented repeatedly to select the optimal parameter combination.

We propose an effective modeling strategy in line with the random lasso, called a recursive random lasso (or elastic net). To efficiently perform high dimensional genomic data analysis, we propose a recursive bootstrap procedure for generating the importance measure and regression modeling. We also propose a novel threshold to effectively select predictor variables in bootstrap regression modeling using a parametric statistical test. Furthermore, a number of candidate predictors, *q*, is also randomly selected in each bootstrap sample (i.e., we do not consider *q* as a tuning parameter). The proposed recursive random lasso (elastic net) is implemented by the following algorithm.

**Algorithm 2** *Recursive random lasso (or elastic net)*

1. Draw *B* bootstrap samples with size *n* by sampling with replacement from the original dataset.
2. For the first bootstrap sample (i.e., *b* = 1), *q* candidate variables are randomly selected and the lasso (or elastic net) is applied for regression modeling. We then obtain estimators ${\hat{\beta }}_{j}^{\left(1\right)}$ for *j* = 1, …, *p*.
3. For *b* ∈ {2, …, *B*}, the importance measure of *x*_*j*_ is calculated as $I_{j}=\left|{\left(b-1\right)}^{-1}\sum_{r=1}^{b-1}{\hat{\beta }}_{j}^{\left(r\right)}\right|$. The *q* candidate variables are randomly selected with a selection probability *I*_*j*_, and the adaptive lasso (or adaptive elastic net) with *w*_*j*_ = 1/*I*_*j*_ is applied for regression modeling. We obtain the estimators ${\hat{\beta }}_{j}^{\left(b\right)}$ for *j* = 1, …, *p*.
4. Final estimators are computed as ${\hat{\beta }}_{j}=B^{-1}\sum_{b=1}^{B}{\hat{\beta }}_{j}^{\left(b\right)}$.
5. Finally, we perform variable selection based on the threshold t* via the parametric statistical test.

#### Parametric Statistical Test for Variable Selection in Bootstrap Regression Modeling (PSTVSboot)

In order to effectively perform feature selection, we propose a parametric statistical test based on the bootstrap regression modeling results. We first consider a *B* × *p* binary matrix **D** obtained from the above recursive bootstrap procedures. We set an element of the binary matrix as *D*_*bj*_ = 1 for a non-zero ${\hat{\beta }}_{j}$ in the *b* ^*th*^ bootstrap sample; otherwise *D*_*bj*_ = 0. In other words, we consider that the binary matrix is obtained from Bernoulli experiments, and let ***D***_*j*_ be a random variable associated with Bernoulli trials as follows:

- $D_{bj}\left({\hat{\beta }}_{j}^{b}\neq 0\right)=1$,
- $D_{bj}\left({\hat{\beta }}_{j}^{b}=0\right)=0$.

The Bernoulli random variable has the following probability density function,

$$\begin{array}{c} f\left(d_{j}\right)=\pi^{d_{j}}{\left(1-\pi \right)}^{1-d_{j}},\;d_{j}=0,1, \end{array}$$ (7)

where the probability *π* can be estimated as follows,

$$\begin{array}{c} \hat{\pi }=\frac{1}{p\times B}\sum_{j=1}^{p}\sum_{b=1}^{B}D_{bj}, \end{array}$$ (8)

which indicates the average of the selection ratio of all predictor variables in *B* bootstrap samples. For reasonable variable selection, we then consider the following statistic:

$$\begin{array}{c} C_{j}=\sum_{b=1}^{B}D_{bj},\;j=1,...,p, \end{array}$$ (9)

which indicates the number of non-zero ${\hat{\beta }}_{j}^{\left(b\right)}$ in *B* Bernoulli trials (i.e. *B* bootstrap samples). The statistic *C*_*j*_ follows the Binomial distribution $b(B,\hat{\pi })$ and has the following probability mass function:

$$\begin{array}{c} f\left(c\right)=\frac{B!}{c!(B-c)!}{\hat{\pi }}^{c}{\left(1-\hat{\pi }\right)}^{B-c},\;c=0,1,...,B. \end{array}$$ (10)

We then calculate a *p*-value for each predictor variable as follows,

$$\begin{array}{cc} \;p-{\text{value}}_{j} & =p\left(c\geq C_{j}|\hat{\pi }\right)\\ & \;=\sum_{c=C_{j}}^{B}\frac{B!}{c!(B-c)!}{\hat{\pi }}^{c}{\left(1-\hat{\pi }\right)}^{B-c}, \end{array}$$ (11)

and finally perform variable selection based on the *p*-value with a threshold t* = 0.05 as follows,

$$\begin{array}{c} {\hat{\beta }}_{j}^{*}={\hat{\beta }}_{j}I\left(p-{\text{value}}_{j}<0.05\right), \end{array}$$ (12)

where *I*(⋅) is an indicator function. We can expect that the parametric statistical test can overcome false positive feature selection results of bootstrap regression modeling. Although we have described the proposed variable selection strategy focused on the random lasso procedure, the parametric statistical test will be a useful tool for bootstrap regression modeling.

## Results

### Monte Carlo Simulations

Monte Carlo simulations were conducted to investigate the effectiveness of the proposed modeling strategy. We simulated 100 datasets from the following linear regression model,

$$\begin{array}{c} y_{i}=x_{i}^{T}\beta +\varepsilon_{i},\;i=1,...,n, \end{array}$$ (13)

where *ε*_*i*_ are *N*(0, *σ* ^2^), and the correlation between *x*_*l*_ and *x*_*m*_ is 0.5^|*l*−*m*|^.

We considered the following simulation situations:

- Type1: *n* = 100 and *p* = 1000 as *β*_*j*_ = 3 for 50 randomly selected variables, otherwise *β*_*j*_ = 0,
- Type2: *n* = 100 and *p* = 1000 as *β*_*j*_ = 3 for 25 randomly selected variables, *β*_*j*_ = −3 for 25 randomly selected variables, otherwise *β*_*j*_ = 0,
- Type3: *n* = 100 and *p* = 1000 as *β*_*j*_ = 3 for 150 randomly selected variables, otherwise *β*_*j*_ = 0.
- Type4: *n* = 100 and *p* = 1000 as *β*_*j*_ = 3 for 75 randomly selected variables, *β*_*j*_ = −3 for 75 randomly selected variables, otherwise *β*_*j*_ = 0,
- Type5: *n* = 50 and *p* = 2000 as *β*_*j*_ = 3 for 40 randomly selected variables, otherwise *β*_*j*_ = 0,
- Type6: *n* = 50 and *p* = 2000 as *β*_*j*_ = 3 for 20 randomly selected variables, *β*_*j*_ = −3 for 20 randomly selected variables, otherwise *β*_*j*_ = 0,
- Type7: *n* = 50 and *p* = 2000 as *β*_*j*_ = 3 for 200 randomly selected variables, otherwise *β*_*j*_ = 0.
- Type8: *n* = 50 and *p* = 2000 as *β*_*j*_ = 3 for 100 randomly selected variables, *β*_*j*_ = −3 for 100 randomly selected variables, otherwise *β*_*j*_ = 0,

To evaluate the proposed recursive random lasso and elastic net procedures, we compared the performance of our methods, recursive random elastic net (RCS.RD.EL), recursive random lasso (RCS.RD.LA), with the lasso (LASSO), adaptive lasso (AD.LA), elastic net (ELA), and existing random lasso (RD.LA). In numerical studies, we used a ridge estimator for weight in the existing adaptive lasso, and we considered the threshold of the existing random lasso to be *s*/*n*, and selected *s* based on the root mean squared error in the validation dataset. We considered the number of bootstrap samples to *B* = 1000 and a dataset constructed with training, validation, and test datasets with sample size *n*, respectively. The tuning parameters were selected by 5-fold cross validation based on the training dataset.

We first evaluated the computational efficiency of our methods. Table 1 shows the computational time required for the existing random lasso in ALGORITHM 1 (RD.LA) and the proposed recursive random lasso in ALGORITHM 2 (RCS.RD.LA). The run time indicates the total time required to estimate the regression model via tuning parameters selection and bootstrap replication. Table 1 shows that the performances of the proposed recursive random lasso is computationally effective compared with the existing random lasso in all simulation situations.

**Table 1:** Running timings for regression modeling for *σ* = 1 via *glmnet* package in R (unit: minute).

	Type 1	Type 2	Type 3	Type 4	Type 5	Type 6	Type 7	Type 8
RCS.RD.LA	14.9	16.1	16.3	16.2	9.2	9.1	8.7	8.7
RD.LA	116.1	123.1	121.6	122.2	58.7	58.9	58.3	58.5

To show the effectiveness of recursive bootstrap strategy, we compared the importance measures for the random lasso procedures. Table 2 shows the average of the importance measures *I*_*j*_ for predictor variables with truly non-zero coefficients and truly zero coefficients in the recursive random elastic net (RCS.RD.EL), recursive random lasso (RCS.RD.LA) and random lasso (RD.LA), where the numbers in parentheses are the average of the importance measures for small number of bootstrap samples *B* = 20.

**Table 2:** Average of importance measures for predictor variables with non-zero and zero coefficients.

		RCS.RD.EL		RCS.RD.LA		RD.LA	
		Non.ZERO	ZERO	Non.ZERO	ZERO	Non.ZERO	ZERO
*σ* = 1	Type1	0.33(0.32)	0.07(0.10)	0.34(0.29)	0.06(0.05)	0.27(0.24)	0.05(0.06)
	Type2	0.29(0.28)	0.06(0.09)	0.30(0.24)	0.05(0.05)	0.24(0.21)	0.05(0.05)
	Type3	0.29(0.33)	0.12(0.19)	0.28(0.22)	0.11(0.09)	0.23(0.21)	0.11(0.01)
	Type4	0.23(0.27)	0.11(0.17)	0.22(0.18)	0.10(0.08)	0.19(0.18)	0.09(0.10)
	Type5	0.08(0.09)	0.02(0.04)	0.08(0.08)	0.02(0.02)	0.05(0.07)	0.02(0.02)
	Type6	0.08(0.09)	0.02(0.04)	0.08(0.08)	0.02(0.02)	0.05(0.07)	0.02(0.02)
	Type7	0.07(0.12)	0.05(0.09)	0.07(0.07)	0.04(0.04)	0.06(0.08)	0.04(0.05)
	Type8	0.07(0.11)	0.05(0.08)	0.06(0.06)	0.04(0.04)	0.05(0.07)	0.04(0.05)
*σ* = 3	Type1	0.33(0.31)	0.07(0.01)	0.34(0.73)	0.06(0.18)	0.26(0.24)	0.05(0.06)
	Type2	0.28(0.29)	0.06(0.09)	0.29(0.26)	0.05(0.05)	0.23(0.20)	0.05(0.05)
	Type3	0.29(0.34)	0.13(0.19)	0.28(0.22)	0.11(0.09)	0.23(0.21)	0.11(0.11)
	Type4	0.22(0.27)	0.11(0.17)	0.21(0.18)	0.10(0.08)	0.18(0.18)	0.09(0.10)
	Type5	0.07(0.09)	0.02(0.04)	0.07(0.08)	0.02(0.02)	0.05(0.07)	0.02(0.02)
	Type6	0.07(0.08)	0.02(0.04)	0.07(0.07)	0.02(0.02)	0.05(0.07)	0.02(0.02)
	Type7	0.07(0.12)	0.05(0.09)	0.07(0.07)	0.04(0.04)	0.06(0.08)	0.04(0.05)
	Type8	0.07(0.11)	0.04(0.08)	0.06(0.06)	0.04(0.04)	0.05(0.07)	0.04(0.05)
*σ* = 9	Type1	0.30(0.32)	0.07(0.11)	0.31(0.26)	0.06(0.05)	0.24(0.22)	0.06(0.06)
	Type2	0.28(0.28)	0.07(0.10)	0.29(0.25)	0.06(0.05)	0.22(0.21)	0.06(0.06)
	Type3	0.29(0.34)	0.13(0.19)	0.27(0.23)	0.11(0.09)	0.23(0.23)	0.11(0.11)
	Type4	0.23(0.27)	0.12(0.16)	0.22(0.18)	0.10(0.08)	0.18(0.18)	0.09(0.10)
	Type5	0.07(0.09)	0.02(0.04)	0.07(0.07)	0.02(0.02)	0.06(0.07)	0.02(0.02)
	Type6	0.07(0.09)	0.02(0.04)	0.07(0.07)	0.02(0.02)	0.05(0.07)	0.02(0.02)
	Type7	0.08(0.12)	0.05(0.10)	0.07(0.07)	0.04(0.05)	0.06(0.08)	0.04(0.05)
	Type8	0.07(0.10)	0.05(0.08)	0.06(0.06)	0.04(0.04)	0.05(0.07)	0.04(0.05)

In the existing random lasso, the importance measure is calculated independently with regression modeling (i.e., in step 1 of ALGORITHM 1). However, in our method, the *I*_*j*_ is recursively calculated during regression modeling. Furthermore, the *I*_*j*_ of our method is based on a randomly selected number of candidate predictor variables *q*, whereas in the existing random lasso method, *I*_*j*_ is based on the tuning parameters *q*_1_ and *q*_2_ selected by minimizing prediction error in the validation dataset. In short, our method provides time-effective procedures compared with the existing random lasso.

From Table 2, it can be seen that the importance measure in our method shows larger differences between truly zero and non-zero coefficients than it does in the existing random lasso, although the difference is small. Furthermore, we can see that the proposed recursive bootstrap procedure also provides the larger differences for importance measure even in the small number of bootstrap samples (i.e., *B* = 20 given in parentheses of Table 2). This implies that the proposed recursive bootstrap approaches perform effectively for feature selection by using the random forest procedure, although our method provides computationally effective modeling results.

**Fig 1:**
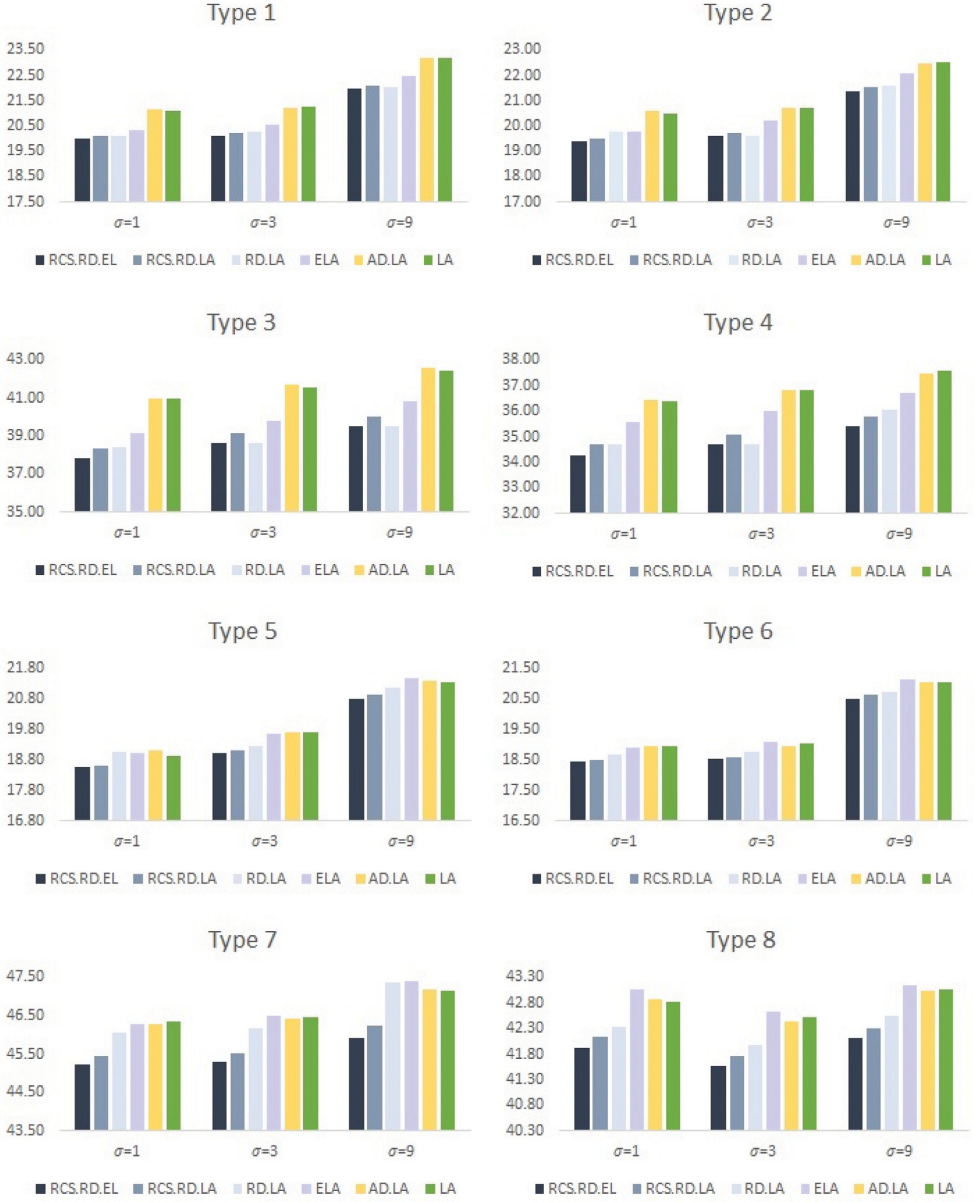
Prediction error: Root mean squared error.

**Fig 2:**
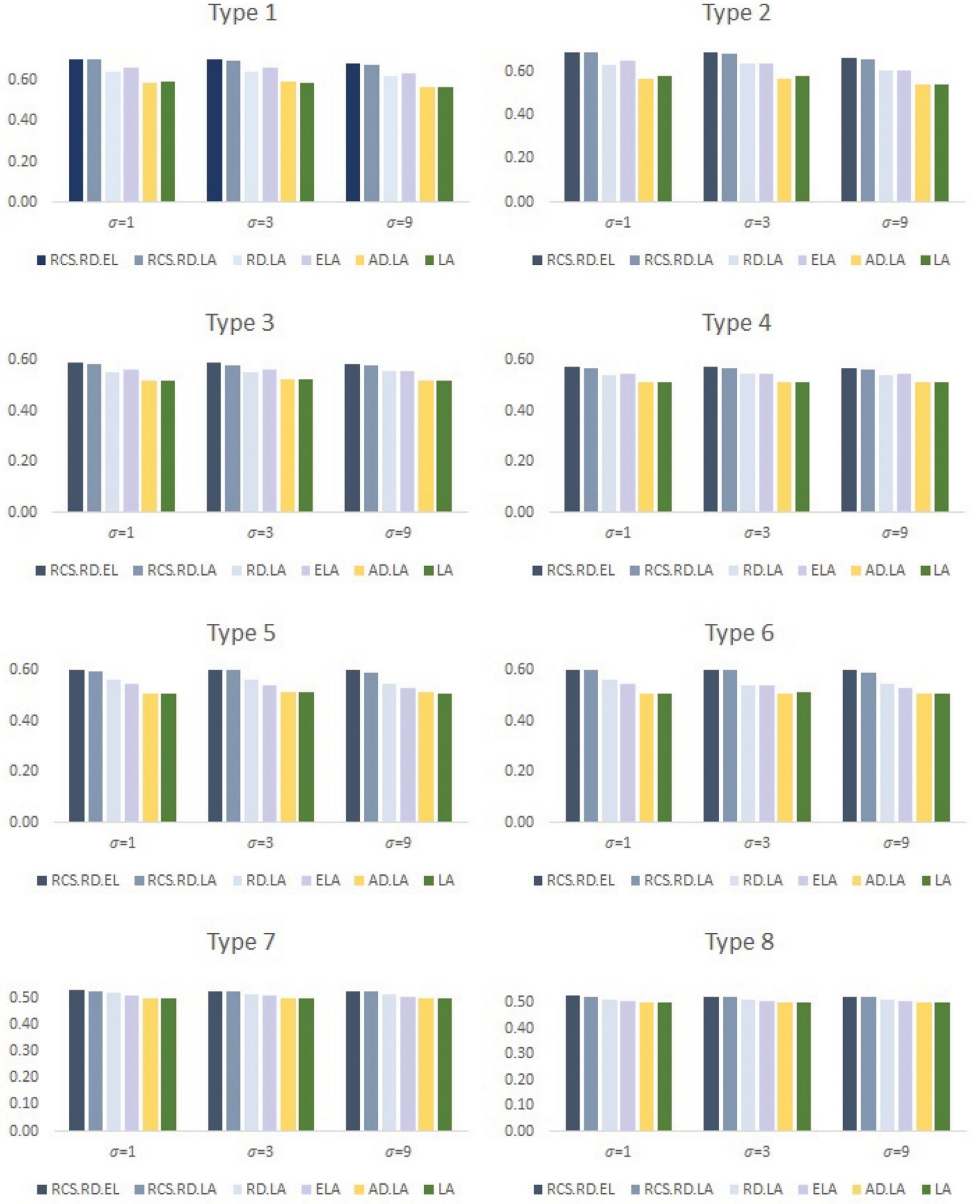
Variable selection results: Average of T.P and T.N.

We then compared the results of regression modeling based on prediction accuracy in the test dataset and the variable selection results shown in Figs 1 and 2.

Fig 1 shows the prediction errors given as average of root mean squared errors using recursive random elastic net (RCS.RD.EL), recursive random lasso (RCS.RD.LA), random lasso (RD.LA), elastic net (ELA), adaptive lasso (AD.LA), and lasso (LASSO). It can be seen though Fig 1 that the proposed recursive random elastic net shows superior prediction accuracy in almost simulation situations. In addition, the proposed recursive random lasso also shows much higher prediction accuracy than the lasso, adaptive lasso or elastic net, and results similar to the existing random lasso, even though the recursive random lasso provides time-effective performances compared with the existing random lasso as shown in Table 1.

We also compared variable selection results given as the average of true positive rate (i.e., the average number of true non-zero coefficients, incorrectly set to zero) and true negative rate (i.e., the average percentage of true zero coefficients, that were correctly set to zero) in Fig 2. We can see though Fig 2 that the proposed recursive random lasso and recursive random elastic net show outstanding performance for variable selection in all simulation situations. On the other hands, the lasso and adaptive lasso show poor results for variable selection in high dimensional data situations, since the methods suffer from the limitation of subset size.

In short, the proposed recursive random lasso and elastic net methods are not only computationally effective but produce outstanding regression modeling results (i.e., prediction accuracy and variable selection). This results imply that our methods can be useful tools for high dimensional genomic alteration data analysis.

### Real World Examples: Identifying Driver Genes of Anti-cancer Drug Sensitivity

We applied the proposed strategies to identify potential driver genes of anti-cancer drug sensitivity in the publicly available “Sanger Genomics of Drug Sensitivity in Cancer dataset from the Cancer Genome Project” (http://www.cancerrxgene.org/). The dataset contains the gene expression levels, copy number and mutation status for 654 cell lines and the half-maximal inhibitory drug concentrations (IC50 values) of 138 anti-cancer drugs as an indicator of drug sensitivity. We considered the expression levels of 13321 genes and the IC50 values of anti-cancer drugs to reveal driver genes, which are available from the resources: “Cell line genetic (mutation and copy number) and gene expression data used for EN analysis” and “Cell line drug sensitivity, mutations and tissue type”, respectively, in “http://www.cancerrxgene.org/”. Many IC50 values are missing from the Sanger dataset, and we therefore considered only 99 anti-cancer drugs, which have non-missing observations for at least 600 cancer cell lines, as response variables. The expression levels of 10% of the genes (i.e., 1332 genes) having the highest variance in all samples were considered as predictor variables. We employed *B* = 1000 bootstrap replications and the tuning parameters were selected by 5-fold cross validation.

To evaluate the proposed methods, we compared the prediction accuracy of the recursive random lasso and elastic net, existing random lasso, elastic net, adaptive lasso and lasso based on 99 regression models corresponding to 99 anti-cancer drugs. Table 3 shows the average of root means squared error of the 99 regression models. We can see through Table 3 that the random lasso type approaches show outstanding performance compared with the *L*_1_-type regularization methods. The proposed recursive random lasso and elastic net show similar performance to the existing random lasso, although our methods show time-effective procedure as shown in the list of run times in Table 3.

**Table 3:** Average of root mean squared errors of 99 regression models and average of running timings (unit: minute).

	RCS.RD.EL	RCS.RD.LA	RD.LA	ELA	AD.LA	LASSO
MSE	1.70	1.70	1.70	1.80	1.74	1.83
Running timings	211.2	32.3	398.9	-	-	-

We then identified potential driver genes using the proposed recursive random elastic net. We focused on five popular anti-cancer drugs: Cisplatin, Docetaxel, Doxorubicin, Gemcitabine and Vinorelbine, which have attracted considerable for cancer research [6, 7]. We will introduce the five anti-cancer drugs.

- **Cisplatin (trade name: Platinol)**: a platinum-compound chemotherapy drug that stops cancer cells from growing. **Target**: DNA crosslinker. **Used to treat**: testicular, ovarian, bladder, head and neck, breast, cervical and prostate cancers. **Side effects**: nausea and vomiting, kidney toxicity, low white blood cell counts, and low red blood cell counts.
- **Docetaxel (trade name: Taxotere)**: belongs to a class of chemotherapy drugs that works by preventing division of cancer cells. **Targets**: Microtubules. **Used to treat**: breast, non-small cell lung, advanced stomach, and head and neck cancers. **Side effects**: nausea, diarrhea, hair loss, nail change, low white blood cell counts, and low red blood cell counts.
- **Doxorubicin (trade name: Adriamycin)**: an anti-cancer chemotherapy drug that is classified as an “anthracycline antibiotic”. It slows or stops the growth of cancer cells, and binds to DNA by intercalation between specific base pairs, thus blocking DNA synthesis [8]. **Target**: DNA intercalation. **Used to treat** leukemia, bladder, breast, stomach, lung, ovarian and thyroid cancers, and soft tissue sarcoma. **Side effects**: hair loss, myelosuppression, oral mucositis, and diarrhea.
- **Gemcitabine (trade name: Gemzar)**: an anti-cancer chemotherapy drug that is classified as an antimetabolite. Gemcitabine prevents the growth of cancer cells, eventually resulting in their destruction. It inhibits thymidylate synthetase, which leads to inhibition of DNA synthesis and cell death [9]. **Targets: DNA replication**.**Used to treat** pancreatic, non-small cell lung, bladder, metastatic breast, and ovarian cancers, and soft-tissue sarcoma. **Side effects**: flu-like symptoms (e.g., muscle pain, fever, headache, etc.), fatigue, and poor appetite.
- **Vinorelbine (trade name: Navelbine)**: an anti-cancer chemotherapy drug that is classified as a “plant alkaloid”. Vinorelbine kills cancer cells by interfering with their DNA, which is necessary for their growth and reproduction. The antitumor activity of vinorelbine is thought to be due primarily to inhibition of mitosis at metaphase through its interaction with tubulin [9]. **Target**: Microtubules. **Used to treat** non-small cell lung, breast, and ovarian cancers, and Hodgkin’s disease. **Side effects**: temporary decrease in white and red blood cells, muscle weakness, and constipation.

We identified the potential driver genes with top 10 largest importance measures *I*_*j*_ among the selected genes for each anti-cancer drug (Table 4). As shown in Table 4, the identified genes are strong candidates for cancer driver genes. This implies that our method provides reliable results for uncovering driver genes. In short, the proposed strategies based on the recursive bootstrap method and parametric statistical test are useful tools for driver gene selection based on high dimensional genomic data analysis.

**Table 4:** Identified potential driver genes of anti-cancer drugs and their evidences.

Drug	Gene	Reference	Disease
Doxorubicin	*TM9SF2*	[10, 11]	Breast carcinoma cells, Colon cancer
	*ENSA*	[12]	Liver, Breast cancers
	*STRAP*	[13]	Colorectal cancer
	*FAT*	[14]	Oral, Breast, Lung, Pancreatic, Gastric cancers
	*VDAC2*	[15]	Muscles of electrically stunned chikens
	*GPM6B*	[16, 17]	Breast, Liver Cancers
	*AMOTL2*	[18]	Ovarian carcinoma
	*IL6ST*	[19]	Lung cancer
	*RPL26-LOC400055*	[20]	Pancreatic cancer
	*NCAM1*	[21]	Lung cancer
Docetaxel	*SLC7A11*	[22, 23]	Breast, Bladder cancers
	*HLA.DQA1*	[24, 25]	Lung squamous cell carcinoma, Breast cancer
	*GOLGA8A*	[26]	Lung cancer
	*CS*	[27]	Pancreatic ductal carcinoma
	*S100A14*	[12, 28]	Esophageal, Ovarian cancers
	*YPEL5*	[29]	Chronic lymphocytic leukemia
	*BTG1*	[30, 31]	Breast, Ovarian cancers
	*KDELR2*	[32]	Breast, Ovarian cancers
	F*KBP1A*	[33]	Breast carcinoma
	*ACTC1*	[34]	Prostate cancer
Gemcitabine	*NEDD9*	[35, 36]	Breast, Lung cancers
	*HEXB*	[37]	Renal carcinoma
	*DDX39*	[38, 39]	Bladder cancer, Lung squamous cell cancer
	*SPOCK1*	[40]	Lung cancer
	*TOB1*	[41, 42]	Breast, Gastric cancers
	*CDH17*	[43, 44]	Gastric cancer
	*PRDX6*	[45]	Lung cancer
	*BAMBI*	[46, 47]	Ovarian, Bladder cancers
	*FST*	[48]	Breast, Ovarian cancers
	*NTS*	[49]	Breast cancer
Vinorelbine	*ZNF706*	[50]	Laryngeal, Head and neck, Gastric cancers
	*TFAP2A*	[51]	Breast cancer
	*PABPC4*	[52]	Breast cancer
	*DFNA5*	[53, 54]	Gastric, Colorectal cancers
	*MGST3*	[55]	Glioblastoma multiforme
	*CD55*	[56]	Prostate cancer
	*CCT5*	[57]	Breast cancers
	*PRDX4*	[58, 59]	Prostate, Lung cancers
	*NDUFC2*	[60]	Ovarian cancer
	*TCP1*	[61]	Colorectal adenocarcinomas
Cisplatin	*CCT3*	[62]	Colorectal cancer
	*IRAK1*	[63]	Colorectal cancer
	*CLIC4*	[64, 65]	Squamous, Ovarian cancers
	*KRT20*	[66]	Colorectal cancer
	*GPI*	[67]	Breast cancer
	*COL4A2*	[68]	Lung Cancer
	*ENC1*	[69, 70]	Colorectal, Colon cancers
	*MRCL3-MRLC2*	[71]	Colorectal cancer
	*TIMP3*	[72, 73]	Prostate, Colorectal cancers
	*MRPS6*	[74, 75]	Parkinson’s disease, Breast cancer

Drug sensitive-specific driver genes were identified by the “Cancer Genome Project”. In the project, they considered regression modeling and applied the elastic net to identify driver genes. The results are given in the project website (http://www.cancerrxgene.org/). There are, however, differences between selected driver genes of our study and given in the project website, since we consider only 10% of genes (i.e., 1332 genes) having the highest variance as candidate genes in regression modeling. Although the identified driver genes by our method are difference from the driver genes identified by the project, we can see through Table 4 that the identified driver genes by our method have strong evidence as cancer driver genes.

**Fig 3:**
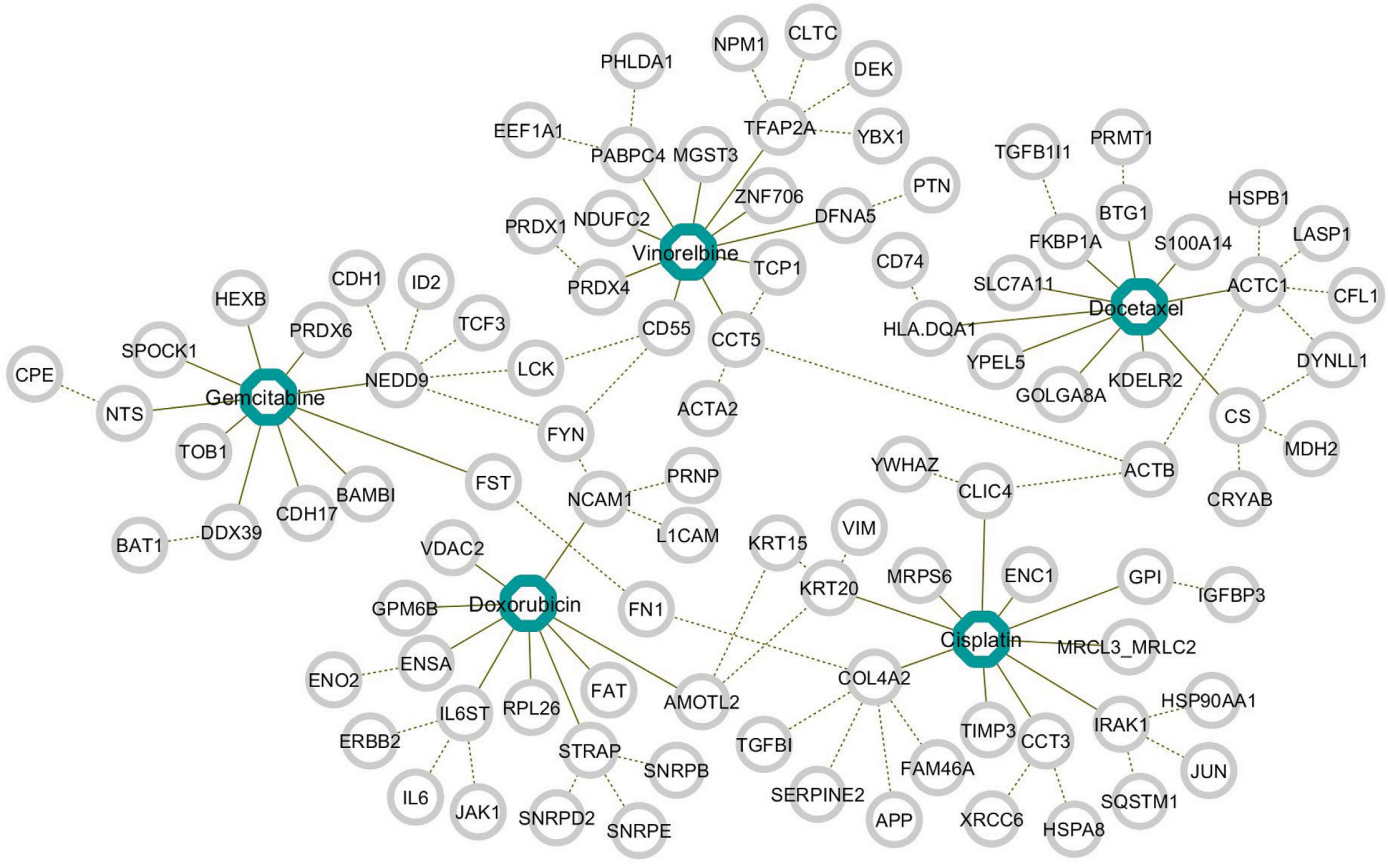
Network for selected driver genes and genes having PPI with identified driver genes.

We also show a gene network based on protein-protein interactions (PPIs). Fig 3 shows the potential driver genes identified in Table 4 as well as genes that have PPIs with the identified genes.

Solid lines indicate potential driver genes identified for each anti-cancer drug and dashed lines indicate PPIs between genes. The anti-cancer drug cisplatin has the largest sub-network constructed by PPIs with a path length of 1. In Fig 3, we can also see that the sub-networks of the five anti-cancer drugs share common genes. The common genes can be considered as driver genes for anti-cancer therapy, and investigation of the common genes may lead to development of effective cancer therapies.

We also focused on driver genes with large sub-network, i.e., *NEDD9, TCP1, CCT5, ACTC1, CS, CLIC4*, and *NCAM1*, these genes are connected with a large number of genes (*n* ≥ 9) by PPIs. Table 5 shows the genes with large sub-networks and their importance measures in the recursive random elastic net.

**Table 5:** Importance measures for gene with large subnetwork.

	Drug	*I*_*j*_	$\text{Ave}I_{j}^{sct}$	$Ave.I_{j}^{all}$
*CCT5*(13)	Vinorelbine	0.0139	0.0028	0.0013
*TCP1*(13)	Vinorelbine	0.0134	0.0028	0.0013
*CS*(13)	Dexorubicin	0.0405	0.0032	0.0015
*ACTC1*(13)	Dexorubicin	0.0347	0.0032	0.0015
*NCAM1*(9)	Docetaxel	0.0204	0.0048	0.0015
*NEDD9*(9)	Gemcitabine	0.1997	0.0160	0.0062
*CLIC4*(10)	Cisplatin	0.0022	0.0039	0.0019

The numbers in parentheses indicate the number of genes connected by PPIs. We can see that the genes with large sub-networks have relatively larger importance measures (*I*_*j*_) than average of all selected genes ($I_{j}^{sct}$) and of all 1332 candidate genes ($I_{j}^{all}$). This implies that possession of a large sub-network can be considered as a crucial feature for predicting anti-cancer drug sensitivity. We can also see through the results that the proposed recursive random elastic net can effectively be used to reveal driver genes with real biological relevance.

## Conclusion

We have proposed a novel statistical strategy based on a recursive bootstrap approach and parametric statistical test (PSTVSboot) for identifying driver genes. To effectively perform high dimensional genomic data analysis, we used recursive bootstrap strategies in line with the random lasso method. Furthermore, we have proposed a parametric statistical test for gene selection based on the results of bootstrap regression modeling.

Numerical studies showed that the proposed methods show outstanding performance for variable selection and prediction accuracy. Furthermore, our methods showed time-effective performance compared with existing random lasso. We expect that our methods based on recursive bootstrap regression modeling and parametric statistical test will be useful tools for high dimensional genomic data analysis, especially driver gene selection. Furthermore, we expected that the proposed parametric test can be used effectively for variable selection in bootstrap regression modeling.

Although the proposed parametric statistical test performs well for feature selection, our method is sensitive to the initial selection of predictor variables, because the initial selection result directly affects the selection probability in the random forest procedure. Thus, further work is required for robust recursive random *L*_1_-type regularization method against initial selection.

Furthermore, we have focused on the proposed recursive random lasso in not theoretical but practical viewpoint. We considered constructing theoretical properties of our method (e.g., consistency of feature selection) as one of further work of this study.

Variation in gene expression levels in cancer is known to be caused by copy number variation, and thus the two features should be considered concurrently when searching for driver genes. We also considered cancer driver gene selection via analysis of copy number driven expression levels via extension of the recursive random lasso strategies.

## References

[1] Tibshirani R (1996) Regression shrinkage and selection via the lasso. J Roy Stat Soc Ser B 73:273–282.

[2] Zou H, Hastie T (2005) Regularization and variable selection via the elastic net. J R Stat Soc Series B 67:301–320. 10.1111/j.1467-9868.2005.00527.x

[3] Wang S, Nam B, Rosset S, Zhu J (2011) Random lasso. Ann Appl Stat 5:468–485. 10.1214/10-AOAS377 22997542 PMC3445423

[4] Zou H, Zhang HH (2009) On the adaptive elastic-net with a diverging number of parameters. Ann Stat 37(4):1733–1751. 10.1214/08-AOS625 20445770 PMC2864037

[5] Hoerl AE, Kennard RW, (1970). Ridge regression: biased estimation for nonorthogonal problems. Techonometrics 12:55–67. 10.1080/00401706.1970.10488634

[6] Gemma A, Li C, Sugiyama Y, Matsuda K, Seike Y, et al (2006) Anticancer drug clustering in lung cancer based on gene expression profiles and sensitivity database. BMC Cancer, 6(174). 10.1186/1471-2407-6-174 PMC1533844 16813650

[7] Tata DB, Hahn G, Dunn F (1993) Ultrasonic absorption frequency dependence of two widely used anti-cancer drugs: doxorubicin and daunorubicin. Ultrasonics 31:447–450. 10.1016/0041-624X(93)90054-4 8236585

[8] Chorawala MR, Oza PM, Shah GB (2012) Mechanisms of Anticancer Drugs Resistance: An Overview. Int J Pharm Sci Drug Res 4:1–9.

[9] DRUG BANK, http://www.drugbank.ca/.

[10] Abou-Sharieha S, Sugii Y, Tuoya Yu D, Chen L, Tokutaka H, Seno M (2009) Identification of TM9SF2 as a candidate of the cell surface marker common to breast carcinoma cells. Chinese J Clin Onco 6:1–9. 10.1007/s11805-009-0001-6

[11] Chiang SF, Tsai MH, Tang R, Hsieh LL, Chiang JM et al (2014) Membrane proteins as potential colon cancer biomarkers: Verification of 4 candidates from a secretome dataset. Surg Sci 5:418–438. 10.4236/ss.2014.510067

[12] Chen YL, Kuo MH, Lin PY, Chuang WL, Hsu CC et al (2013) ENSA expression correlates with attenuated tumor propagation in liver cancer. Biochem Biophys Res Commun 442:56–61. 10.1016/j.bbrc.2013.10.165 24211627

[13] Buess M, Terracciano L, Reuter J, Ballabeni P, Boulay JL, et al (2004) STRAP is a strong predictive marker of adjuvant chemotherapy benefit in colorectal cancer. Neoplasia 6:813–820. 10.1593/neo.04307 15720808 PMC1531685

[14] Katoh M (2012) Function and cancer genomics of FAT family genes. Int J Oncol 41:1913–1918. 10.3892/ijo.2012.1669 23076869 PMC3583642

[15] Samah NA, Amid A, Yisof F (2011) Over expression of Voltage Dependent Anion Channer 2 (VDAC2) in muscles of electrically stuunned chickens. IIUM Eng J 12:213–222.

[16] Bilecova-Rabajdova M, Urban P, Gregova K, Varga J, Fialkovicova V, et al (2014) Breast carcinoma progression and tumour vascular markers related to apoptotic mechanisms. Dis Markers 2014 10.1155/2014/156034 24696529 PMC3948469

[17] Stefanska B, Bouzelmat A, Huang J, Suderman M, Hallett M, et al (2013) Discovery and validation of DNA hypomethylation biomarkers for liver cancer using HRM-specific probes. PLoS ONE 8(8):e68439 10.1371/journal.pone.0068439 23950870 PMC3737236

[18] Kohn KW, Zeeberg BR, Reinhold WC, Sunshine M, Luna A, et al (2012) Gene expression profiles of the NCI-60 human tumor cell lines define molecular interaction networks governing cell migration processes. PLoS ONE 7(5). 10.1371/journal.pone.0035716 PMC3343048 22570691

[19] Sun L, Sui L, Cong X, Ma K, Ma X, et al (2014) Low incidence of IL6ST (gp130) mutations in exon 6 in lung cancer of a Chinese cohort. Cancer Genetics 207:291–298. 10.1016/j.cancergen.2014.07.003 25242236

[20] Li C, Ge M, Yin Y, Luo M, Chen Daijie (2012) Silencing expression of ribosomal protein L26 and L29 by RNA interfering inhibits proliferation of human pancreatic cancer PANC-1 cells. Mol Cell Biochem, 370:127–139. 10.1007/s11010-012-1404-x 22868929

[21] Kashiwagi K, Ishii J, Sakaeda M, Arimasu Y, Shimoyamada H, et al (2012) Differences of molecular expression mechanisms among neural cell adhesion molecule 1, synaptophysin, and chromogranin A in lung cancer cells. Pathol Int 62:232–245. 10.1111/j.1440-1827.2011.02781.x 22449227

[22] Drayton RM, Dudziec E, Peter S, Bertz S, Hartmann A, et al (2014) Reduced expression of miRNA-27a modulates cisplatin resistance in bladder cancer by targeting the cystine/glutamate exchanger SLC7A11. Clin Cancer Res 20:1990–2000. 10.1158/1078-0432.CCR-13-2805 24516043 PMC3974662

[23] Liu XX, Li XJ, Zhang B, Liang YJ, Zhou CX, et al (2011) MicroRNA-26b is underexpressed in human breast cancer and induces cell apoptosis by targeting SLC7A11. FEBS Letters 585:1363–1367. 10.1016/j.febslet.2011.04.018 21510944

[24] Kohno T, Kunitoh H, Mimaki S, Shiraishi K, Kuchiba A, et al (2011) Contribution of the TP53, OGG1, CHRNA3, and HLA-DQA1 genes to the risk for lung squamous cell carcinoma1. J Thorac Oncol 6:813–817. 21623257 10.1097/JTO.0b013e3181ee80ef

[25] Spraggs CF, Budde LR, Briley LP, Bing N, Cox CJ, et al (2011) HLA-DQA1*02:01 is a major risk factor for lapatinib-induced hepatotoxicity in women with advanced breast cancer. J Clin Oncol 29:667–673. 10.1200/JCO.2010.31.3197 21245432

[26] Kim BY, Lee J, Park SJ, Bang OS, Kim NS (2013) Gene expression profile of the A549 human non-small cell lung carcinoma cell line following treatment with the seeds of descurainia sophia, a potential anticancer drug. Evid Based Complement Alternat Med 2013:584604 10.1155/2013/584604 23935669 PMC3712200

[27] Gaude E, Frezza C. (2014) Defects in mitochondrial metabolism and cancer. Gaude and Frezza Cancer & Metabolism 2(10). 10.1186/2049-3002-2-10 PMC4108232 25057353

[28] Cho H, Shin HY, Kim S, Kim SY, Chung JY, et al (2014) The role of S100A14 in epithelial ovarian tumors. Oncotarget 5:3482–3496. 10.18632/oncotarget.1947 24939856 PMC4116497

[29] Velusamy T, Palanisamy N, Kalyana-Sundaram S, Sahasrabuddhe AA, Maher CA, et al (2013) Recurrent reciprocal RNA chimera involving YPEL5 and PPP1CB in chronic lymphocytic leukemia. Proc Natl Acad Sci U S A 110:3035–3040. 10.1073/pnas.1214326110 23382248 PMC3581970

[30] Zhao Y, Gou WF, Chen S, Takano Y, Xiu YL, Zheng HC. (2013) BTG1 expression correlates with the pathogenesis and progression of ovarian carcinomas. Int J Mol Sci 14:19670–19680. 10.3390/ijms141019670 24084718 PMC3821579

[31] Zhu R, Zou ST, Wan JM, Li W, Li XL, et al (2013) BTG1 inhibits breast cancer cell growth through induction of cell cycle arrest and apoptosis. Oncol Rep 30:2137–2144. 23982470 10.3892/or.2013.2697

[32] Jarzab M, Dudaladava V, Simek K (2005) Comparison of the expression profile in breast cancer and ovarian cancer. Breast Cancer Res 7.

[33] Bhushan L, Kandpal RP (2011) EphB6 receptor modulates micro RNA profile of breast carcinoma cells. PLoS ONE 6(7). 10.1371/journal.pone.0022484 PMC3139643 21811619

[34] Huang HC, Zheng S, VanBuren V, Zhao Z (2010) Discovering disease-specific biomarker genes for cancer diagnosis and prognosis. echnol Cancer Res Treat 9:219–230. 10.1177/153303461000900301 20441232

[35] Jin Y, Li F, Zheng C, Wang Y, Fang Z, et al (2014) NEDD9 promotes lung cancer metastasis through epithelial-mesenchymal transition. Int J Cancer 134:2294–2304. 10.1002/ijc.28568 24174333

[36] Kong C, Wang C, Wang L, Ma M, Niu C, et al (2011) NEDD9 is a positive regulator of epithelial-mesenchymal transition and promotes invasion in aggressive breast cancer. PLoS ONE 6(7):e22666 10.1371/journal.pone.0022666 21829474 PMC3145662

[37] Okochi T, Seike H, Higashino K, Hada T, Watanabe S, et al (1979) Alterationof HexosaminidaseIsozymesin Human Renal Carcinoma. Cancer Research 39:1829–1834. 427815

[38] Kato M, Wei M, Yamano S, Kakehashi A, Tamada S, et al (2012) DDX39 acts as a suppressor of invasion for bladder cancer. Cancer Sci 103:1363–1369. 10.1111/j.1349-7006.2012.02298.x 22494014 PMC7659185

[39] Sugiura T, Nagano Y, Noguchi Y (2007) DDX39, upregulated in lung squamous cell cancer, displays RNA helicase activities and promotes cancer cell growth. Cancer Biol Ther 6:957–964. 10.4161/cbt.6.6.4192 17548965

[40] Miao L, Wang Y, Xia H, Yao C, Cai H, et al (2013) SPOCK1 is a novel transforming growth factor-*β* target gene that regulates lung cancer cell epithelial-mesenchymal transition. Biochem Biophys Res Commun 440:792–797. 10.1016/j.bbrc.2013.10.024 24134845

[41] Jiao Y, Ge CM, Meng QH, Cao JP, Tong J, et al (2007) Adenovirus-mediated expression of Tob1 sensitizes breast cancer cells to ionizing radiation. Acta Pharmacol Sin 28:1628–1636. 10.1111/j.1745-7254.2007.00647.x 17883950

[42] Kundu J, Wahab SM, Kundu JK, Choi YL, Erkin OC, et al (2012) Tob1 induces apoptosis and inhibits proliferation, migration and invasion of gastric cancer cells by activating Smad4 and inhibiting *β*-catenin signaling. Int J Oncol 41:839–848. 10.3892/ijo.2012.1517 22710759 PMC3582759

[43] Lee HJ, Nam KT, Park HS, Kim MA, Lafleur BJ, et al (2010) Gene expression profiling of metaplastic lineages identifies CDH17 as a prognostic marker in early stage gastric cancer. Gastroenterology 139:213–225. 10.1053/j.gastro.2010.04.008 20398667 PMC2917327

[44] Zhang J, Liu QS, Dong WG. (2011) Blockade of proliferation and migration of gastric cancer via targeting CDH17 with an artificial microRNA. Med Oncol 28:494–501. 10.1007/s12032-010-9489-0 20393816

[45] Yun HM, Park KR, Lee HP, Lee DH, Jo M, et al (2014) PRDX6 promotes lung tumor progression via its GPx and iPLA2 activities. GFree Radic Biol Med 69:367–376. 10.1016/j.freeradbiomed.2014.02.001 24512906

[46] Khin SS, Kitazawa R, Win N, Aye TT, Mori K, et al (2009) BAMBI gene is epigenetically silenced in subset of high-grade bladder cancer. Int. J. Cancer 125:328–338. 10.1002/ijc.24318 19326429

[47] Pils D, Wittinger M, Petz M, Gugerell A, Gregor W, et al (2010) BAMBI is overexpressed in ovarian cancer and co-translocates with Smads into the nucleus upon TGF-beta treatment. Gynecol Oncol 117:189–197. 10.1016/j.ygyno.2009.12.034 20189233

[48] Sengupta D, Bhargava DK, Dixit A, Sahoo BS, Biswas S, et al (2014) ERR*β* signalling through FST and BCAS2 inhibits cellular proliferation in breast cancer cells. Br J Cancer 110:2144–2158. 10.1038/bjc.2014.53 24667650 PMC3992508

[49] Dupouy S, Viardot-Foucault V, Alifano M, Souaze F, Plu-Bureau G, et al (2009) The neurotensin receptor-1 pathway contributes to human ductal breast cancer progression. PLoS ONE 4(1):e4223 10.1371/journal.pone.0004223 19156213 PMC2626627

[50] Colombo J, Provazzi PJS, Calmon MF, Pires LC, Rodrigues NC, et al (2013) Expression, purification and molecular analysis of the human ZNF706 protein. Biol Proced Online 15 10.1186/1480-9222-15-10 24060497 PMC3848911

[51] Berlato C, Chan KV, Price AM, Canosa M, Scibetta AG, et al (2011) Alternative TFAP2A isoforms have distinct activities in breast cancer. Breast Cancer Res 13 10.1186/bcr2838 21375726 PMC3219183

[52] Kostianets O, Antoniuk S, Filonenko V, Kiyamova R. (2012) Immunohistochemical analysis of medullary breast carcinoma autoantigens in different histological types of breast carcinomas. Diagn Pathol 7:161 10.1186/1746-1596-7-161 23181716 PMC3533517

[53] Akino K, Toyota M, Suzuki H, Imai T, Maruyama R, et al (2007) Identification of DFNA5 as a target of epigenetic inactivation in gastric cancer. Cancer Sci 98:88–95. 10.1111/j.1349-7006.2006.00351.x 17083569 PMC11158324

[54] Yokomizo K, Harada Y, Kijima K, Shinmura K, Sakata M, et al (2012) Methylation of the DFNA5 gene is frequently detected in colorectal cancer. Anticancer Res 32:1319–1322. 22493364

[55] Chen QR, Hu Y, Yan C, Buetow K, Meerzaman D (2014). Systematic genetic analysis identifies Cis-eQTL target genes associated with glioblastoma patient survival. PLoS ONE 9(8). 10.1371/journal.pone.0105393 PMC4136869 25133526

[56] Loberg RD, Day LL, Dunn R, Kalikin LM, Pienta KJ (2006) Inhibition of decay-accelerating factor (CD55) attenuates prostate cancer growth and survival in vivo. Neoplasia 8:69–78. 10.1593/neo.05679 16533428 PMC1584292

[57] Ooe A, Kato K, Noguchi S. (2007). Possible involvement of CCT5, RGS3, and YKT6 genes up-regulated in p53-mutated tumors in resistance to docetaxel in human breast cancers. Breast Cancer Res Treat 101:305–315. 10.1007/s10549-006-9293-x 16821082

[58] Schulte J (2011) Peroxiredoxin 4: a multifunctional biomarker worthy of further exploration. BMC Medicine 9 10.1186/1741-7015-9-137 22196027 PMC3260115

[59] Ummanni R, Barreto F, Venz S, Scharf C, Barett C, et al (2012) Peroxiredoxins 3 and 4 are overexpressed in prostate cancer tissue and affect the proliferation of prostate cancer cells in vitro. J Proteome Res 11:2452–2466. 10.1021/pr201172n 22424448

[60] Chen Y, McGee J, Chen X, Doman TN, Gong X, et al (2014) Identification of druggable cancer driver genes amplified across TCGA datasets. PLoS ONE 9(5). 10.1371/journal.pone.0098293 PMC4038530 24874471

[61] Coghlin C, Carpenter B, Dundas SR, Lawrie LC, Telfer C, et al (2006) Characterization and over-expression of chaperonin t-complex proteins in colorectal cancer. J Pathol 210:351–357. 10.1002/path.2056 16981251

[62] Chung FH, Chen HD, Lee HC, Lee HC. Integration of network theory with gene expression data on disease progression significantly improves prediction for human colorectal cancer biomarkers http://sansan.phy.ncu.edu.tw/ hclee/ppr/HFNCC8.4.pdf

[63] Zhiying X, Zhou P, Zhou PZ, Zhang J, Cao LB, et al (2013) miR-142-3p inhibits LPS-induced activation of NF-kB by targeting IRAK1 in colorectal cancer. Centr Eur J Immunol 38:416–420. 10.5114/ceji.2013.39755

[64] Suh KS, Mutoh M, Gerdes M, Yuspa SH (2005) CLIC4, an Intracellular Chloride Channel Protein, Is a Novel Molecular Target for Cancer Therapy. J Investig Dermatol Symp Proc 10:105–109. 10.1111/j.1087-0024.2005.200402.x 16358817

[65] Yao Q, Qu X, Yang Q, Wei M, Kong B (2009) CLIC4 mediates TGF-beta1-induced fibroblast-to-myofibroblast transdifferentiation in ovarian cancer. Oncol Rep 22:541–548. 19639201 10.3892/or_00000469

[66] Chan CW, Wong NA, Liu Y, Bicknell D, Turley H, et al (2009) Gastrointestinal differentiation marker Cytokeratin 20 is regulated by homeobox gene CDX1. Proc Natl Acad Sci U.S.A. 106:1936–1941. 10.1073/pnas.0812904106 19188603 PMC2644142

[67] Wu G, Guo Z, Chatterjee A, Huang X, Rubin E, et al (2006) Overexpression of glycosylphosphatidylinositol (GPI) transamidase subunits phosphatidylinositol glycan class T and/or GPI anchor attachment 1 induces tumorigenesis and contributes to invasion in human breast cancer. Cancer Res 66(20):9829–36. 10.1158/0008-5472.CAN-06-0506 17047043

[68] Edwards YJK, Beecham GW, Scott WK, Khuri S, Bademci G, et al (2011) An integrated expression profiling reveals target genes of TGF-b and TNF-a possibly mediated by microRNAs in lung cancer cells. PLoS ONE 8(2):e56587. 10.1371/journal.pone.0056587 PMC3577886 23437179

[69] Amaia G, Ana F, Ruben A, Inaki I, Ziortza I, et al (2007) Gene expression model for the classification of human colorectal cancer and potential CRC biomarkers search in Drug Discovery Technology, London.

[70] Larriba MJ, Gonzalez-Sancho JM, Barbachano A, Niell N, Ferrer-Mayorga G, et al (2013) Vitamin D Is a Multilevel Repressor of Wnt/b-Catenin Signaling in Cancer Cells. Cancers (Basel) 5:1242–1260. 10.3390/cancers5041242 24202444 PMC3875938

[71] Llarena AM, Garcia A, Suarez B, Jangi M, Garrido P, et al (2009) Gene expression profile of human colorectal cancer using oligonucleotide microarray. J Clin Oncol 27.

[72] Lin H, Zhang Y, Wang H, Xu D, Meng X, et al (2012) Tissue inhibitor of metalloproteinases-3 transfer suppresses malignant behaviors of colorectal cancer cells. Cancer Gene Ther 19 845–851. 10.1038/cgt.2012.70 23037807

[73] Shinojima T, Yu Q, Huang SK, Li M, Mizuno R, et al (2012) Heterogeneous epigenetic regulation of TIMP3 in prostate cancer. Epigenetics 7:1279–1289. 10.4161/epi.22333 23023649 PMC3499329

[74] Iwao-Koizumi K, Matoba R, Ueno N, Kim SJ, Ando A, et al (2005) Prediction of docetaxel response in human breast cancer by gene expression profiling. J Clinical Oncology 23:422–431. 10.1200/JCO.2005.09.078 15659489

[75] Papapetropoulos S, Ffrench-Mullen J, McCorquodale D, Qin Y, Pablo J, et al (2006) Multiregional gene expression profiling identifies MRPS6 as a possible candidate gene for Parkinson’s disease. Gene Expr 13:205–215. 10.3727/000000006783991827 17193926 PMC6032441

